# Feeding time index and mortality in intensive care stroke patients: an exploratory cohort study

**DOI:** 10.55730/1300-0144.6140

**Published:** 2025-01-05

**Authors:** Nevzat Mehmet MUTLU, Duygu Karaköse ÇALIŞKAN, Tülay Tunçer PEKER, Özlem Balkız SOYAL, Ayşe Pınar TİTİZ, Behiye Deniz KOSOVALI, Göksal GÜNERHAN, Büşra TEZCAN

**Affiliations:** 1 Department of Critical Care, University of Health Sciences, Ankara Bilkent City Hospital, Ankara, Turkiye; 2 Department of Anesthesiology and Reanimation - Critical Care, University of Health Sciences, Gülhane Education and Research Hospital, Ankara, Turkiye; 3 Department of Anesthesiology and Reanimation, University of Health Sciences, Ankara Bilkent City Hospital, Ankara, Turkiye; 4 Department of Neurology, University of Health Sciences, Ankara Bilkent City Hospital, Ankara, Turkiye; 5 Department of Neurosurgery, University of Health Sciences, Ankara Bilkent City Hospital, Ankara, Turkiye; 6 Department of Anesthesiology and Reanimation - Critical Care, Ankara Etlik City Hospital, Ankara, Turkiye

**Keywords:** Feeding Time Index [non-MeSH], ratio of feeding days to total intensive care unit length of stay [non-MeSH], intensive care units, stroke, mortality, oral feeding

## Abstract

**Background/aim:**

Stroke is a major cause of morbidity and mortality worldwide, with especially high mortality in intensive care unit (ICU) patients, whose prognosis is worsened by dysphagia, malnutrition, and aspiration risk. The Feeding Time Index (FTI)—defined as the ratio of feeding days to total ICU length of stay—quantified nutritional exposure. This exploratory cohort study aimed to evaluate the association between FTI and mortality in ICU patients with acute ischemic stroke (IS) or hemorrhagic stroke (HS) and to examine the potential role of FTI, together with the Acute Physiology and Chronic Health Evaluation II (APACHE II) score and oral feeding status, as a supportive metric for quantitative assessment of nutritional practices.

**Materials and methods:**

In this retrospective observational study, 239 stroke patients admitted to the ICU were analyzed. Demographic data, clinical characteristics, APACHE II scores, time to feeding initiation, feeding routes, and FTI values were collected. Univariate and multivariate Cox regression analyses were performed to identify independent predictors of mortality.

**Results:**

Among 239 ICU patients, 56.9% had IS and 43.1% had HS, with an ICU mortality rate of 31%. Higher FTI values were significantly associated with a lower mortality risk in univariate Cox analysis (hazard ratio (HR) = 0.06), but this association was not maintained in the multivariate model. In contrast, inability to feed orally (HR = 14.8) and higher APACHE II scores (HR = 1.07) remained the main independent predictors of mortality.

**Conclusion:**

These findings suggest that, while FTI is a useful complementary and exploratory indicator of the relationship between feeding duration and clinical outcomes in ICU stroke patients, mortality prediction relies more heavily on clinical factors such as the APACHE II score and oral feeding status. Thus, FTI should be viewed as a supportive metric for quantitatively monitoring nutritional practices, and further multicenter prospective studies controlling more thoroughly for disease severity are needed to clarify its prognostic value and role in guiding nutritional strategies.

## 1. Introduction

Stroke is defined as the sudden death of brain cells due to oxygen deprivation, occurring within 24 h of symptom onset or thereafter, resulting from the blockage or rupture of a cerebral artery without an apparent extracerebral vascular cause [1–3]. It is typically characterized by sudden weakness or numbness, usually affecting one side of the face, arm, or leg [4].

Of all strokes, 80%–87% are ischemic in origin, while the remainder are hemorrhagic [2]. In Türkiye, the annual incidence of stroke is approximately 125,000, with 65.1% classified as ischemic and 34.9% as hemorrhagic [5]. Hemorrhagic stroke (HS) carries a higher mortality rate and tends to occur earlier than ischemic stroke (IS), in which mortality more commonly occurs in the later period [2,5,6].

Globally, stroke accounts for a substantial proportion of all deaths and is the second leading cause of mortality. More than two-thirds of stroke-related deaths occur in developing countries [4,7]. In developed countries, recent reductions in stroke-associated hospitalizations and mortality rates have been attributed to improved acute stroke care [2].

High rates of morbidity and mortality in stroke patients are largely attributed to factors such as dysphagia (with reported incidences of up to 90%), aspiration risk, nutrition-related complications, and marked declines in quality of life [8,9]. In patients with stroke monitored in the intensive care unit (ICU), early enteral nutrition and properly managed nutritional support have been shown to decrease rates of infection, hospital and ICU stay duration, and mortality [10,11].

Malnutrition in stroke patients increases the risk for infections, pressure ulcers, early mortality, impaired functional outcomes, slower recovery, and delayed rehabilitation, as demonstrated in various studies [8,9]. For stroke patients unable to eat orally, sustained enteral feeding via percutaneous endoscopic gastrostomy (PEG) or nasogastric tube (NGT) is recommended to prevent malnutrition [10]. However, there is no consensus regarding the optimal timing for initiating nutrition or the preferred enteral feeding method [8].

In ICU patients, optimal timing and duration of nutrition are critically important for improving clinical prognosis. Early enteral feeding has been shown in several studies to reduce infection rates, the duration of hospital and ICU stay, and mortality [10,11]. Nevertheless, no specific study or guideline has investigated the evaluation of feeding duration as an index—defined by the ratio of feeding days to ICU length of stay—in the literature.

The hypothesis herein was that, in patients with acute IS and HS monitored in the ICU, the Feeding Time Index (FTI; the ratio of feeding days to total ICU length of stay) may be associated with mortality, and that the FTI can be regarded not as a primary independent prognostic marker, but as a complementary parameter reflecting the duration of nutritional exposure.

The aim of this study was to explore, within an exploratory cohort design, the relationship between the FTI and mortality in patients with acute IS and HS monitored in the ICU, and investigate the potential utility of FTI—alongside established clinical indicators such as the Acute Physiology and Chronic Health Evaluation II (APACHE II) score and oral feeding status—as a supportive tool for the quantitative evaluation of nutritional practices.

## 2. Materials and methods

### 2.1. Study design and participants

Following approval from the hospital ethics committee (Approval number: E-19-2471), data from 330 patients admitted to general ICUs with neurological diagnoses between June 2016 and June 2018 were retrospectively evaluated. After the patients were grouped according to diagnosis, only those with HS or IS were included in the study, forming a cohort of 239 patients. Those admitted to the ICU due to traumatic brain injury, intracranial mass, hypoxic brain injury, dementia or Alzheimer’s disease, seizure disorders, or progressive muscular diseases were excluded.

Demographic and clinical characteristics of stroke patients—including age, sex, length of stay in the ICU, APACHE II score and associated expected mortality rate, number of comorbidities, specific comorbidities (hypertension (HT), diabetes mellitus (DM), coronary artery disease (CAD), and chronic kidney disease (CKD)), day of nutrition initiation, ratio of feeding duration to total ICU stay (FTI), need for NGT, need for PEG, number of patients able to feed orally, duration of nasogastric (NG) feeding, day of PEG initiation, duration of PEG feeding, duration of oral feeding, feeding routes while in the ICU, nutritional status at discharge, overall mortality rate, and need for nonfatal cardiopulmonary resuscitation (CPR) during hospitalization—were recorded. Patients were categorized into IS and HS groups, as well as survivor and nonsurvivor groups, with comparisons made between their demographic and clinical features.

The following variables were considered potential risk factors for mortality and were assessed for their impact on mortality: sex, age, time to initiation of nutrition, number of comorbidities, HT, DM, CAD, CKD, stroke type (HS or IS), FTI, need for NGT, need for CPR, oral nutrition, and APACHE II score.

**Figure 1:**
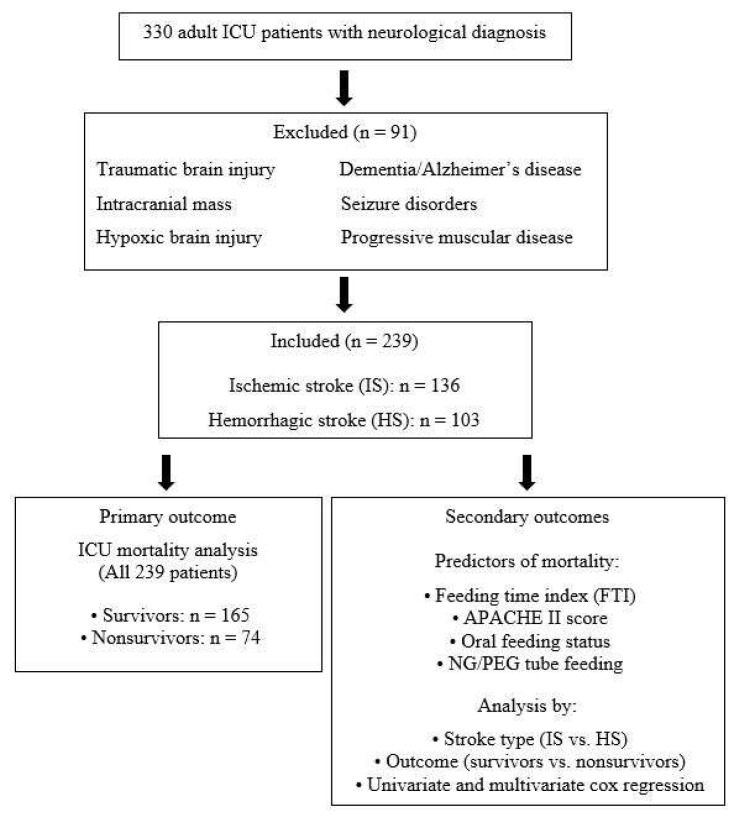
Flow diagram of patient selection and the analysis.

The flow diagram of the patient selection and analysis is presented in Figure 1.

### 2.2. Statistical analysis

Statistical analyses of the data obtained in the study were performed using IBM SPSS Statistics for Windows 26.0 (IBM Corp., Armonk, NY, USA). Continuous variables were expressed as the mean ± standard deviation (SD). The normality of the distribution of numerical data was assessed using the Shapiro–Wilk test. For comparison of normally distributed numerical data, the Student’s t test was used, with results evaluated assuming equality of variances. For numerical data not conforming to normal distribution, the Mann–Whitney U test was employed. Categorical data were presented as numbers and percentages, and the Pearson chi-squared test was used for group comparisons. Logistic regression analysis was performed to identify factors affecting mortality, and p < 0.05 was considered statistically significant.

## 3. Results

Data from 239 patients were analyzed. The mean age of the cohort was 67.59 ± 14.81 years, and 55.6% (n = 133) were male. Of the patients, 136 (56.9%) were diagnosed with IS and 103 (43.1%) were diagnosed with HS. During ICU follow-up, 74 patients (31%) died. The most prevalent comorbidity was HT (49.8%), the mean FTI was 0.88, and the most commonly used feeding route was oral nutrition (48.5%). Additional descriptive statistics for the stroke patients are presented in Table 1.

Comparisons of nutritional, sociodemographic, and clinical features between the HS and IS patients are shown in Table 2. Both the mean age and ICU length of stay were significantly higher in the IS group than in the HS group (p = 0.049 and p = 0.004, respectively; Table 2). The sex distribution between these groups also differed significantly (p = 0.022, Table 2), with patients in the HS group more frequently being male. The mortality rate was significantly higher in the HS group compared to the IS group (p = 0.022, Table 2). No significant differences were found in the other clinical characteristics between the groups (p > 0.05, Table 2).

**Figure 2:**
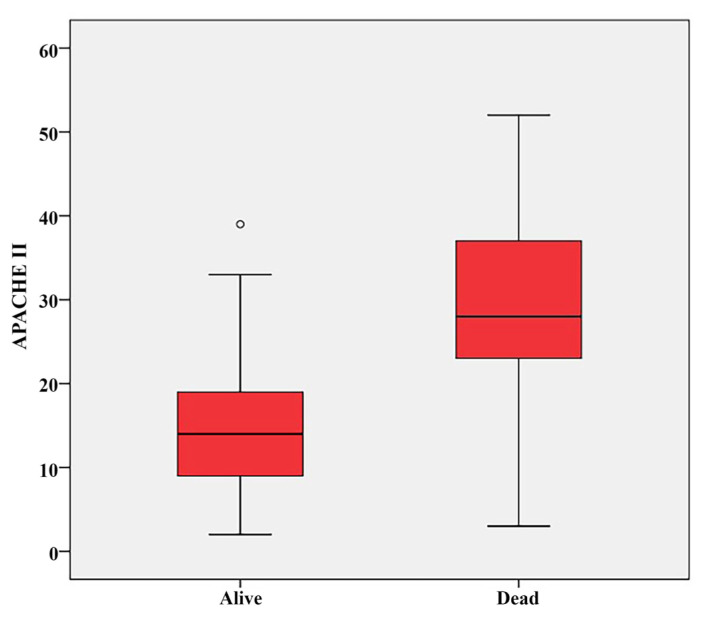
Box plot of the distribution of APACHE II scores according to ICU mortality results.

Comparisons of nutritional, sociodemographic, and clinical characteristics between the survivors and nonsurvivors are presented in Table 2. The groups were statistically similar in terms of age (p = 0.439) and sex ratio (p = 0.539; Table 2). Survivors had significantly longer ICU stays, higher FTI values, longer duration of NGT feeding, and longer oral feeding durations than nonsurvivors (p < 0.001, p = 0.013, p < 0.001, and p = 0.004, respectively; Table 2). APACHE II scores and expected mortality percentages were significantly lower among survivors (p < 0.001 and p < 0.001, respectively; Figure 2, Table 2). Nonsurvivors exhibited a higher prevalence of CKD, greater need for nonfatal CPR during ICU stay, and greater need for NGT feeding (respectively, p = 0.003, p < 0.001, and p < 0.001, respectively; Table 2). The proportion of patients able to receive oral nutrition was significantly lower among nonsurvivors (p < 0.001, Table 2). The distribution of nutritional support routes also differed significantly (p < 0.001, Table 2): the survivors were more likely to be fed solely by oral or oral plus NG methods, while the nonsurvivors were more often fed exclusively via NGTs. No other clinical characteristics showed significant differences between the groups (p > 0.05, Table 2).

The results of univariate and multivariate Cox regression analyses for risk factors affecting mortality, along with hazard ratios (HRs) and 95% confidence intervals (CIs) for statistically significant parameters, are presented in Table 3. In the univariate analysis, sex, age, time to initiation of feeding, number of comorbidities, HT, DM, and CAD were not statistically significant (p = 0.683, p = 0.866, p = 0.154, p = 0.266, p = 0.059, p = 0.635, and p = 0.886, respectively; Table 3). Stroke type, need for CPR, FTI, need for NG, oral feeding status, CKD, and APACHE II score were statistically significant in the univariate analysis (p = 0.005, p < 0.001, p < 0.001, p < 0.001, p < 0.001, p = 0.023, and p < 0.001, respectively; Table 3). The corresponding HRs were stroke type 1.93 (95% CI: 1.22–3.06), need for CPR 5.16 (95% CI: 2.86–9.31), need for NGT 63.2 (95% CI: 9.53–419.2), oral feeding 32.2 (95% CI: 11.7–88.2), CKD 2.01 (95% CI: 1.10–3.68), and APACHE II score 1.11 (95% CI: 1.09–1.13). FTI had a protective effect against mortality, with an HR of 0.06 (95% CI: 0.02–0.19). Variables not statistically significant in the univariate model were not entered into the multivariate model.

In the multivariate model, the stroke type, FTI, need for NGT, HT, and CKD were not statistically significant (p = 0.118, p = 0.774, p = 0.823, p = 0.604, and p = 0.601, respectively). However, the need for CPR, oral feeding status, and APACHE II score remained statistically significant (p = 0.032, p < 0.001, and p < 0.001, respectively; Table 3). The HRs for these factors were 1.92 (95% CI: 1.06–3.47) for the need for CPR, 14.8 (95% CI: 5.15–42.3) for inability to feed orally, and 1.07 (95% CI: 1.04–1.09) per one-point increase in APACHE II score. Thus, mortality risk was higher in patients requiring CPR, in those unable to feed orally, and with increasing APACHE II scores, as reflected by the corresponding HRs.

Kaplan–Meier survival curves comparing survival according to the need for CPR and oral feeding status in ICU are presented in Figures 3A and 3B, respectively. According to the log-rank test results comparing the survival distributions, patients who did not require CPR, had significantly greater survival probability than those requiring CPR (p < 0.001, Figure 3A). Similarly, patients able to feed orally exhibited significantly higher survival probability compared to those unable to do so (p < 0.001, Figure 3B).

## 4. Discussion

This retrospective study systematically evaluated nutritional, demographic, and clinical factors that may affect mortality in patients with HS and IS monitored in general ICUs. The findings revealed that nutritional parameters, stroke type, need for CPR, and clinical severity indices, such as the APACHE II score, more strongly determine mortality than demographic factors.

Effect of the stroke type and demographic characteristics on mortality: The overall reported mortality rate (31%) among the stroke patients herein is consistent with previously reported ICU mortality rates for stroke patients [12,13]. Additionally, the mortality rate in patients with HS was significantly higher than that in patients with IS. However, the lack of significance of the stroke type in the multivariate analysis suggests that mortality is primarily due to complications. The literature indicates that the overall prognosis of IS is better than that of HS, where death occurs especially in the acute and subacute phases [14]. The higher mortality observed in HS has been attributed to the increased prevalence of severe complications such as a sudden rise in intracranial pressure, cerebral edema, and herniation in these patients [4,12,13]. The increased mortality in the HS group indicates that surgical interventions and targeted blood pressure management protocols may be important in reducing mortality in this population, highlighting the need for multicenter studies with larger patient populations.

In contrast to Salvadori et al. [14], who found no difference in the sex distribution between HS and IS patients, the higher proportion of males in the HS group herein may have been related to the greater prevalence among men of risk factors such as HT and smoking [15]. As observed in previous studies, sex did not have a significant impact on mortality in the current cohort [16].

The higher mean age in the IS patients compared to the HS patients (69.5 vs. 65) was likely associated with the increased prevalence of atherosclerotic risk factors in older age [14,17]. However, the lack of a significant relationship between age and mortality suggests that mortality in ICU stroke patients may depend more on acute clinical parameters than demographic characteristics [16,18]. The insignificant effect of age and sex on mortality reinforces the notion that intensive therapeutic protocols in ICU settings have a greater impact than demographic factors. Similarly, large multicenter studies have shown that age and sex alone do not determine mortality among stroke patients in the ICU [18].

Influence of nutritional parameters on mortality: In this study, oral feeding status emerged as one of the strongest determinants of mortality. However, it should also be considered that this association may reflect a strong confounding effect closely related to underlying disease severity and neurological status rather than nutrition itself. The 14.8-fold increase in mortality risk among stroke patients unable to feed orally suggests that this variable may function less as an indicator of an independent nutritional intervention and more as a surrogate marker of clinically critical conditions. These include severe neurological deficits, impaired consciousness, and a high propensity for aspiration. Therefore, although the findings demonstrate that inability to feed orally is strongly associated with mortality, this relationship should not be interpreted as causal, nor should it be concluded that mortality can be reduced solely by intervening on the feeding route. Instead, the results indicate that oral feeding status primarily reflects an integrated risk profile related to disease severity, the degree of neurological recovery, and the risk of complications.

In the univariate analysis, the need for NGT feeding was associated with a 63.2-fold increase in mortality risk, reflecting the more fragile clinical condition of these patients. The higher mortality rate observed in patients receiving NGT feeding is attributed to more severe neurological deficits and swallowing disorders. The loss of significance in the multivariable model supports the notion that NGT feeding is more likely to represent a strong indicator of disease severity rather than an independent risk factor. From this perspective, both the inability to feed orally and the need for an NGT should be regarded not as independent interventional targets related to the route of nutrition, but as surrogate markers reflecting severe neurological injury, dysphagia, impaired consciousness, and the consequent burden of complications. Consistent with this view, the literature has also shown that mortality and morbidity are increased in stroke patients who are unable to take food orally and must receive enteral nutrition via NGT; this increase is largely explained by underlying neurological deterioration, dysphagia, and an elevated risk of aspiration [19,20]. The high mortality observed in patients dependent on an NGT may be an indicator of serious neurological complications such as dysphagia or impaired consciousness. Moreover, compared with patients who are able to feed orally, these individuals are at higher risk for aspiration pneumonia [8,20–22]. An NGT may lead to the accumulation of secretions in the hypopharynx, a reduced cough reflex, and impaired airway protection. In addition, the presence of the tube may increase colonization of the pharyngeal region with gastric bacteria [8].

Early placement of PEG within the first two weeks after stroke onset has been shown to effectively support nutritional and hydration needs, since many patients with dysphagia experience long-term difficulties with oral feeding. Although mortality is high in the initial months following PEG insertion, long-term survivors may regain a reasonable level of functional capacity [9]. However, a similar confounding issue is present here as well; the indication for PEG is most often established in patients with more severe neurological injury who are anticipated to be unable to maintain oral intake for a prolonged period, so the elevated mortality observed may be related more to the high-risk profile of the selected patient group than to the PEG procedure itself. Therefore, the association of both NGT and PEG use with mortality should be interpreted by carefully considering the strong confounding effects of disease severity and neurological status. Prospective randomized studies with detailed control of disease severity are needed to determine whether early PEG placement or swallowing rehabilitation actually has been associated with reduced mortality in patients with impaired oral intake.

In conclusion, although the data obtained in this study demonstrate that variables related to the route of nutrition (ability to feed orally, need for NGT/PEG) are strongly associated with mortality, they also suggest that these variables may essentially function as surrogate markers representing a complex clinical picture driven by disease severity and neurological status. Accordingly, the findings should be interpreted within a cautious framework that avoids direct causal claims, centers on potential confounding effects related to disease severity, and positions nutritional parameters primarily as adjunct clinical indicators to aid prognostic assessment rather than as standalone causal determinants.

In the univariate analysis, the FTI appeared to have a protective effect against mortality (HR: 0.06), suggesting that the duration of nutritional exposure during ICU stay may be associated with clinical outcomes. Although previous studies on ICU patients have evaluated factors such as calorie intake, timing of nutrition initiation, and feeding duration with respect to survival and morbidity [23,24], none have specifically examined the impact of the FTI on in-hospital mortality among ICU stroke patients. Malnutrition is known to suppress immune function and increase the risk of infectious complications [25,26], whereas adequate enteral nutrition has been shown to accelerate neurological and cognitive recovery, reduce complications, and decrease mortality [3]. Nevertheless, the FTI is essentially a purely time-based metric—the ratio of the number of days with nutrition to the total ICU length of stay—and does not directly reflect true nutritional adequacy, as it excludes qualitative nutritional components such as caloric and protein intake and individual requirements. Moreover, because the FTI is structurally influenced by both ICU length of stay and survival time, it is subject to the risk of immortal time bias [27], characterized by the fact that patients who survive longer can, by definition, accumulate more feeding days; consequently, higher FTI values are more likely among survivors, and the FTI–mortality association observed in univariate analyses should not be interpreted as causal but rather as partly driven by a guarantee-time effect and residual confounding. In summary, findings based on the FTI and any inferences regarding adequate nutrition must be interpreted cautiously and within a restricted framework, with careful consideration of disease severity (e.g., APACHE II score) and the time-dependent nature of this index. Within this context, the FTI should be regarded as a complementary metric for the quantitative monitoring of malnutrition and nutritional practices in ICU stroke patients, rather than as an independent prognostic marker, and should be viewed as an adjunctive tool that complements clinically stronger predictors of mortality such as APACHE II score and oral feeding status.

Prognostic value of clinical parameters and resuscitation need: Herein, each unit increase in APACHE II score was associated with a 7% increased risk of mortality in stroke patients, reaffirming the prognostic reliability of this score in ICU populations including stroke patients [16,28–32]. APACHE II scores have shown significant predictive value for mortality even when assessed alongside radiological and clinical data in stroke cohorts. APACHE II is widely used as it offers a comprehensive assessment of the patient’s acute physiological state and comorbidities. Since it is calculated using the worst values from the first day of admission, the score is independent of treatment and insensitive to differences in care quality. A high APACHE II score is associated with pathologies such as multiple organ failure and systemic inflammation [16,28,31]. Effective use of mortality indices such as the APACHE II in the ICU provides valuable prognostic information for clinical decision-making [16].

Moreover, the 1.92-fold increased risk of mortality in patients needing in-hospital CPR reflects the fatal impact of hypoxic brain injury and postresuscitation syndrome following cardiac arrest. Few studies have explored prognosis after in-hospital cardiac arrest, with most guidelines based on out-of-hospital scenarios. The limited literature on survival after in-hospital CPR in ICU patients supports our findings, reporting very poor survival rates and high mortality in this population. Longer resuscitation duration has been linked to lower survival [33–36], with multiple organ failure most commonly the cause of death [37,38]. Due to the retrospective nature of this study, detailed data on CPR duration and frequency were unavailable.

CKD emerged as a significant factor in univariate analysis but lost significance in the multivariate model, likely due to its effect being accounted for by other clinical variables (e.g., the APACHE II) and its relatively smaller impact on mortality.

The introduction of the FTI in this study provides an original and complementary approach to quantitatively monitoring nutritional practices in stroke patients treated in the ICU. The FTI enables the patient-specific nutrition process to be quantified, thereby allowing more detailed and comparable data on nutritional exposure during ICU care and facilitating the assessment of the integration of nutritional protocols into clinical practice.

However, the fact that only the APACHE II score and inability to feed orally emerged as independent determinants of mortality in the multivariable analysis indicates that these clinical parameters play a more dominant role than the FTI in prognostic assessment. Therefore, the FTI should be considered not as a standalone independent prognostic tool for predicting patient outcomes in the ICU, but rather as an adjunctive metric that complements strong clinical indicators such as the APACHE II score and oral feeding status.

This study has several limitations. First, the single-center, retrospective design limits the generalizability of the findings to other centers and larger populations. Retrospective data collection prevented full standardization of nutritional programs, protocols, and feeding routes, which may have introduced bias into comparisons of feeding durations and methods. The FTI captures only the duration of nutrition and does not include qualitative aspects such as calorie intake, nutrient composition, or individual requirements; therefore, its impact on clinical outcomes cannot be fully clarified. Only the ICU length of stay was evaluated; the absence of postdischarge follow-up data restricts conclusions to the intensive care period. The FTI is a time-dependent variable directly determined by ICU length of stay and survival time, creating a risk of immortal time bias due to potential misclassification of time-dependent exposures. This structural limitation would be better addressed in future prospective studies using methods that appropriately model time-dependent covariates (e.g., time-dependent Cox models) [39].

## 5. Conclusion

In this study, the relationship between the FTI and mortality was examined in patients with acute IS and HS monitored in the ICU. The findings indicate that the FTI is significantly associated with mortality only in univariate analyses, whereas it does not remain an independent predictor in the multivariate Cox regression model. In contrast, inability to feed orally and higher APACHE II scores emerged as the main clinical determinants associated with mortality in the multivariable analysis.

Therefore, the FTI is more appropriately considered a supportive, exploratory indicator of feeding duration in relation to mortality, rather than a primary or independent prognostic factor. Although lower mortality was observed at the univariate level among patients with higher FTI values—suggesting that the duration and organization of nutrition during the ICU stay may be important—this association should not be regarded as independent of disease severity and other clinical variables.

This research demonstrated that the FTI provides a practical and complementary metric for quantitatively monitoring nutritional practices in ICU stroke patients, while clinical indicators such as the APACHE II score and oral feeding status play a more dominant role in predicting mortality. To more clearly define the prognostic value of the FTI and its place in guiding nutritional strategies, multicenter prospective studies incorporating more detailed adjustment for disease severity and using appropriate time-dependent analyses are warranted.

## Figures and Tables

**Figure 3A:**
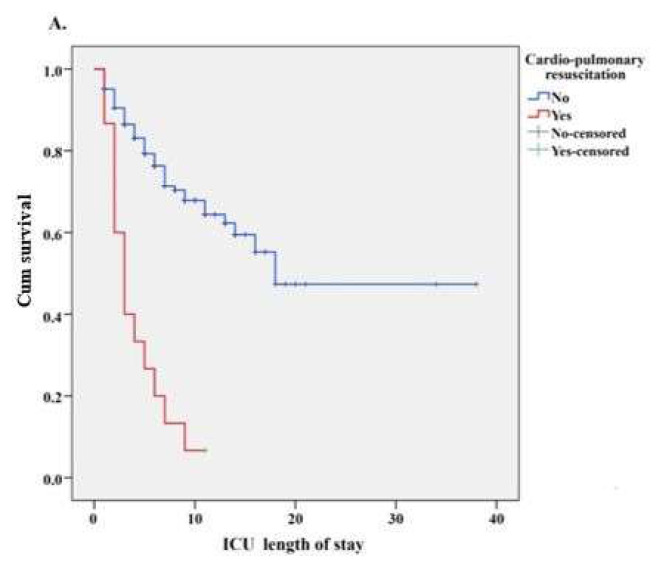
Comparison of ICU length of stay for CPR application during intensive care follow-up with Kaplan–Meier survival curves.

**Figure 3B:**
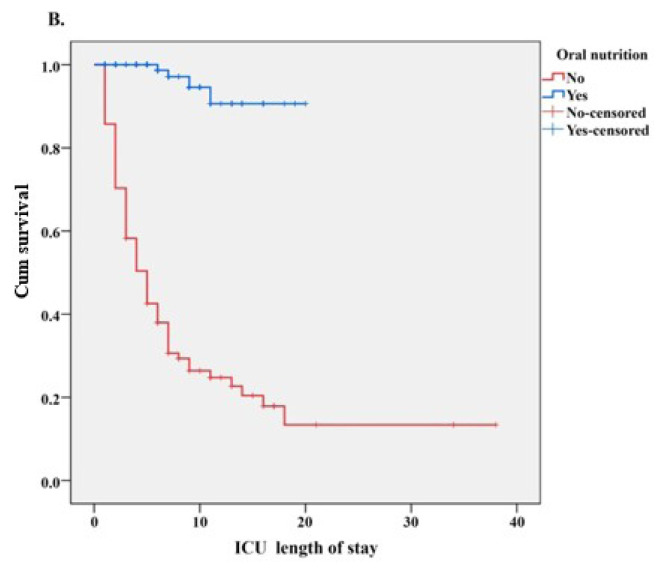
Comparison of ICU length of stay with Kaplan–Meier survival curves according to the oral nutritional status.

**Table 1:** Evaluation of the characteristics of stroke patients followed in the general ICU.

	Patients with stroke (n = 239)
**Age**	69 (21–95) (67.59 ± 14.81)
**Sex**	
Male	133 (55.6%)
Female	106 (44.4%)
**APACHE II**	17 (2–52) (19.10 ± 10.93)
**ICU length of stay (days)**	5 (1–38) (6.45 ± 5.12)
**Mortality**	74 (31%)
**Diagnostic groups**	
IS	136 (56.9%)
HS	103 (43.1%)
**Number of comorbidities**	1 (0–5) (1.13 ± 1.04)
**Comorbidities**	
HT	119 (49.8%)
CAD	61 (25.5%)
DM	52 (21.8%)
CKD	22 (9.2%)
**Number of patients who underwent CPR**	15 (6.3%)
**Feeding start day**	2 (1–4) (1.77 ± 0.74)
**FTI**	0.88 (0.25–1) (0.83 ± 0.17)
**Nutritional features**	
Need for NGT	119 (49.8%)
NGT feeding time	4 (1–19) (4.35 ± 3.65)
Need for PEG	9 (3.8%)
PEG opening day	6 (1–24) (7.44 ± 8)
PEG feeding time	12 (3–15) (10.22 ± 4.32)
**Nutritional route during ICU stay**	
Oral	116 (48.5%)
NG	82 (34.3%)
Oral + NG	32 (13.4%)
PEG	4 (1.7%)
PEG + NG	5 (2.1%)
**Nutritional pattern at discharge** (n=165)	
NG	11 (6.7%)
PEG	10 (6.1%)
Oral	144 (87.3%)

HS: Hemorrhagic stroke, IS: ischemic stroke, NG: nasogastric, PEG: percutaneous endoscopic gastrostomy tube, HT: hypertension, DM: diabetes mellitus, CAD: coronary artery disease, CKD: chronic kidney disease, CPR: cardio-pulmonary resuscitation, FTI: Feeding Time Index (ratio of feeding time to ICU length of stay).

**Table 2:** Comparison of patients with HS and IS and stroke patients who lived and died.

	HS (n = 103)	IS (n = 136)	p-value	Dead (n = 74)	Alive (n = 165)	p-value
**Age**	68 (24–93) (65 ± 16.22)	71.5 (21–95) (69.56 ± 13.37)	**0.049** a	72 (28–95) (68.97 ± 14.84)	69 (21–93) (66.98 ± 14.80)	0.439^a^
**Sex**						
Male	66 (64.1%)	67 (49.3%)	**0.022** b	39 (52.7%)	94 (57%)	0.539^b^
Female	37 (35.9%)	69 (50.7%)		35 (47.3%)	71 (43%)	
**ICU length of stay (days)**	4 (1–38) (5.65 ± 5.14)	6 (1–34) (7.05 ± 5.04)	**0.004** a	3 (1–18) (4.51 ± 3.63)	6 (1–38) (7.32 ± 5.45)	**<0.001** a
**APACHE II**	18 (2–51) (19.87 ± 11.73)	17 (2–52) (18.51 ± 10.29)	0.462^a^	28 (3–52) (30.24 ± 9.95)	14 (2–39) (14.10 ± 6.95)	**<0.001** a
**Expected mortality %**	29.13 (3.8–98) (38.51 ± 29.81)	26.21(3.8–98.3) (34 ± 25.58)	0.535^a^	63.9 (4.4–98.3) (65.33 ± 23.88)	18.65 (3.8–89.9) (22.83 ± 16.79)	**<0.001** a
**Mortality**	40 (38.8%)	34 (25%)	**0.022** b	**-**	**-**	**-**
**Number of comorbidities**	1 (0–4) (0.98 ± 1.01)	1 (0–5) (1.24 ± 1.05)	0.051^a^	1 (0–5) (1.27 ± 1.17)	1 (0–3) (1.06 ± 0.98)	0.293^a^
**Comorbidities**						
HT	46 (44.7%)	73 (53.7%)	0.167^b^	31 (41.9%)	88 (53.3%)	0.102^b^
DM	19 (18.4%)	33 (24.3%)	0.280^b^	17 (23%)	35 (21.2%)	0.760^b^
CAD	22 (21.4%)	39 (28.7%)	0.199^b^	19 (25.7%)	42 (25.5%)	0.971^b^
CKD	6 (5.8%)	16 (11.8%)	0.116^b^	13 (17.6%)	9 (5.5%)	**0.003** b
**Number of patients who underwent CPR**	8 (7.8%)	7 (5.1%)	0.408^b^	14 (18.9%)	1 (0.6%)	**<0.001** c
**Diagnostic groups**						
HS	-	-	-	40 (54.1%)	63 (38.2%)	**0.022** b
IS	-	-		34 (45.9%)	102 (61.8%)	
**Feeding start day**	2 (1–4) (1.77 ± 0.75)	2 (1–4) (1.77 ± 0.74)	0.883^a^	2 (1–4) (1.76 ± 0.84)	2 (1–4) (1.78 ± 0.70)	0.489^a^
**FTI**	0.86 (0.33–1) (0.81 ± 0.18)	0.89 (0.25–1) (0.85 ± 0.17)	0.200^a^	0.80 (0.25–1) (0.77 ± 0.22)	0.90 (0.50–1) (0.86 ± 0.14)	**0.013** a
***Nutritional features***						
Need for NGT	50 (48.5%)	69 (50.7%)	0.737^b^	74 (100%)	45 (27.3%)	**<0.001** b
NGT feeding time	3 (1–19) (3.82 ± 3.40)	4 (1–17) (4.74 ± 3.79)	0.150^a^	2 (1–17) (3.39 ± 2.95)	5 (1–19) (5.93 ± 4.14)	**<0.001** a
Need for PEG	4 (3.9%)	5 (3.7%)	1.000^c^	0 (0%)	9 (5.5%)	0.060^c^
PEG opening day	5 (1–24) (8.75 ± 10.46)	6 (1–17) (6.40 ± 6.54)	0.730^a^	-	6 (1–24) (7.44 ± 8)	-
PEG feeding time	11 (4–14) (10 ± 4.54)	12 (3–15) (10.40 ± 4.66)	1.000^a^	-	12 (3–15) (10.22 ± 4.32)	-
**Number of patients who are able to feed orally**	58 (56.3%)	90 (66.2%)	0.120^b^	4 (5.4%)	144 (87.3%)	**<0.001** b
**Oral feeding time**	3 (1–14) (4.45 ± 3.81)	5 (1–20) (5.09 ± 3.55)	0.120^a^	1 (1–1) 1 ± 0	4 (1–20) (4.94 ± 3.65)	**0.004** a
**Nutritional route during ICU stay**						
Oral	51 (49.5%)	65 (47.8%)	0.077^c^	0 (0%)	116 (70.3%)	**<0.001** c
NG	41 (39.8%)	41 (30.1%)		70 (94.6%)	12 (7.3%)	
Oral + NG	7 (6.8%)	25 (18.4%)		4 (5.4%)	28 (17%)	
PEG	2 (1.9%)	2 (1.5%)		0 (0%)	4 (2.4%)	
PEG + NG	2 (1.9%)	3 (2.2%)		0 (0%)	5 (3%)	
**Nutritional route at discharge**						
NG	3 (4.8%)	8 (7.8%)	0.770^c^	-	11 (6.7%)	**-**
Oral	56 (88.9%)	88 (86.3%)		-	144 (87.3%)	
PEG	4 (6.3%)	6 (5.9%)		-	10 (6%)	

a Mann–Whitney test with median (min–max),

b Ki-kare test,

c Fisher’s Exact test.

**Table 3:** Results of univariate and multivariate Cox regression analysis performed to determine risk factors affecting ICU mortality.

	Univariate		Multivariate	
	p-value	HR (CI 95%)	p-value	HR (CI 95%)
**Sex** Female / male	0.683	-	ni	-
**Age**	0.866	-	ni	-
**Time to start feeding**	0.154	-	ni	-
**Number of comorbidities**	0.266	-	ni	-
**HT** yes / no	0.059	-	ni	-
**DM** yes / no	0.635	-	ni	-
**CAD** yes / no	0.886	-	ni	-
**CKD** yes / no	**0.023** ^*^	2.01 (1.10–3.68)	ns	-
**Diagnostic type** HS / IS	**0.005** ^*^	1.93 (1.22–3.06)	ns	-
**FTI**	**<0.001** ^*^	0.06 (0.02–0.19)	ns	-
**NG need** yes / no	**<0.001** ^*^	63.2 (9.53–419.2)	ns	-
**CPR application during hospitalization** yes / no	**<0.001** ^*^	5.16 (2.86–9.31)	**0.032** ^*^	1.92 (1.06–3.47)
**Oral nutrition** no / yes	**<0.001** ^*^	32.2 (11.7–88.2)	**<0.001** ^*^	14.8 (5.15–42.3)
**APACHE II**	**<0.001** ^*^	1.11 (1.09–1.13)	**<0.001** ^*^	1.07 (1.04–1.09)

ni: Not included in the multivariate model, ns: not significant in the multivariate model, HR: hazard ratio, CI: confidence interval.
